# Occurrence, genetic diversity, and recombination of maize lethal necrosis disease-causing viruses in Kenya

**DOI:** 10.1016/j.virusres.2020.198081

**Published:** 2020-09

**Authors:** Francis M. Mwatuni, Aggrey Bernard Nyende, Joyce Njuguna, Xiong Zhonguo, Eunice Machuka, Francesca Stomeo

**Affiliations:** a International Maize and Wheat Improvement Center (CIMMYT), P.O. Box 1041 – 00621, Nairobi, Kenya; b Jomo Kenyatta University of Agriculture and Technology, P.O. Box 62000-00100, Nairobi, Kenya; c Kenya Plant Health Inspectorate Service(KEPHIS), P.O. Box 49592-00100, Nairobi, Kenya; d Biosciences Eastern and Central Africa, International Livestock Research Institute (BecA – ILRI) Hub, P.O. Box 30709-00100, Nairobi, Kenya; e University of Arizona, Tucson, AZ, 85721, USA

**Keywords:** ABCF, African Biosciences Challenge Fund, CDFA, Phytosanitary Certification Manual, CIMMYT, International Maize and Wheat Improvement Center, KEPHIS, Kenya Plant Health Inspectorate Service, MCMV, Maize chlorotic mottle virus, MLN, Maize lethal necrosis, NPPOs, National Plant Protection Organizations, SCMV, Sugarcane mosaic virus, Surveillance, Maize lethal necrosis, Maize chlorotic mottle virus, Sugarcane mosaic virus, Recombination, Kenya

## Abstract

Maize is the most important food crop in Kenya accounting for more than 51 % of all staples grown in the country. Out of Kenya’s 5.3 million ha total crops area, more than 2.1 million ha is occupied by maize which translates to 40 % of all crops area. However, with the emergence of maize lethal necrosis (MLN) disease in 2011, the average yields plummeted to all-time lows with severely affected counties recording 90–100% yield loss in 2013 and 2014. The disease is mainly caused by *Maize chlorotic mottle virus* (MCMV) in combination with *Sugarcane mosaic virus* (SCMV) or other potyviruses. In this study, a country-wide survey was carried out to assess the MLN causing viruses in Kenya, their distribution, genetic diversity, and recombination. The causative viruses of MLN were determined by RT-PCR using virus-specific primers and DAS-ELISA. Next-generation sequencing (NGS) data was generated, viral sequences identified, genetic diversity of MLN viruses was determined, and recombination was evaluated. MCMV and SCMV were detected in all the maize growing regions at varying levels of incidence, and severity while MaYMV, a polerovirus was detected in some samples through NGS. However, there were some samples in this study where only MCMV was detected with severe MLN symptoms. SCMV Sequences were highly diverse while MCMV sequences exhibited low variability. Potential recombination events were detected only in SCMV explaining the elevated level of diversity and associated risk of this virus in Kenya and the eastern Africa region.

## Highlights

• *Sugarcane mosaic virus* (SCMV) and *Maize chlorotic mottle virus* (MCMV) were the only MLN causing viruses detected.
• MCMV and SCMV are widely distributed in all maize growing regions in Kenya.
• SCMV was more diverse and MCMV was largely conserved with little genetic diversity.
• MCMV alone can lead to severe MLN development.
• Genetic viral recombination signals detected only in SCMV viral genome samples.

## 1 Introduction

Maize (*Zea mays L.*) is the third most important crop in the world and has become an integral part of modern society. Over 4.5 billion people use it as a staple food and as animal feed (FAO, 2016). The annual yield of maize in Kenya in 2016 was 3.39 million tons, and its value exceeded $65 billion (FAO-STAT, 2016). However, with the emergence of maize lethal necrosis (MLN) disease in the country in 2011, the maize yield losses have increased significantly compared to the yield loss recorded from the combination of all other biotic and abiotic factors (Wangai et al., 2012). Yield losses due to MLN in Kenya in the marketing year 2014/2015 was estimated to be up to 10 %, which translated to over 50 million US dollars (USDA Gains report, 2015). A community survey assessment on the distribution and impact of MLN in Kenya in 2013 revealed that the disease affected 22 % of maize produced that year, which translated to about 187 million US dollars in losses (De Groote et al., 2016). Nearly all commercial maize varieties in Kenya have been confirmed to be susceptible to MLN, both under natural and artificial infection in studies done in 2012–2013 (Prasanna, 2015; Semagn et al., 2015; Marenya et al., 2018).

MLN in Kenya is caused by double infection of maize plants with *Maize chlorotic mottle virus* (MCMV), a *Machlomovirus* (Nutter et al., 1989; Lommel et al., 1991a), and *Sugarcane mosaic virus* (SCMV), a *Potyvirus* (Jones et al., 2007; Adams et al., 2012). However, MLN can also be caused by MCMV in synergy with other potyviruses, namely *Maize dwarf mosaic virus* (MDMV) in the genus *Potyvirus*, *Wheat streak mosaic virus* (WSMV) in the genus *Tritimovirus* (Pruss et al., 1997), or the recently described *Johnsongrass mosaic virus* (JGMV) also in the genus *Potyvirus* (Stewart et al., 2014, 2017). Any potyvirus that infects maize can potentially interact synergistically with MCMV to cause MLN (Adams et al., 2012). The possibility of MCMV to combine with other native potyviruses of cereals poses a big challenge to maize production in Kenya. Amongst these potyviruses, SCMV is more dominant in East Africa while MDMV and JGMV have been reported only in Kenya (Wangai et al., 2012; Mahuku et al., 2015a; and Stewart et al., 2017). WSMV is not known to occur in Africa but is found in North America, South America, Europe and Australia (Hadi et al., 2011).

SCMV was first recorded to infect maize by Brandes (1920) in the USA. In Eastern Africa, SCMV was reported in sugarcane (McDonald, 1936; Wallace, 1937; Hansford, 1935) and in maize (Riley, 1960). Later, SCMV was found to occur in all maize growing regions in Kenya, Uganda and Tanzania (Kulkarni, 1973). Louie in his survey confirmed the presence of SCMV in 20 districts (now counties) of the Central highlands, Rift valley highlands and western regions of Kenya (Louie, 1980). Since then, SCMV has been endemic in Kenya and described as the main MLN causative agent in coinfection with MCMV in most studies of MLN emergence in eastern Africa (Adams et al., 2014; Mahuku et al., 2015a,b and Wamaitha et al., 2018).

Maize lethal necrosis has spread fast in the eastern and central Africa region after its first report in Kenya (Wangai et al., 2012). For instance, in Tanzania, the disease was first reported in 2012 in regions around Lake Victoria and Arusha (CIMMYT Periodic Newsletter, Dec 2012). MLN was also reported in Uganda in 2012 in the Kenya border districts of Busia and Tororo (CIMMYT Periodic Reports Newsletters, Dec 2012) and has been detected in eastern Uganda districts of Iganga and Mbale (Kagoda et al., 2016). In Rwanda, it was first reported in 2013 and was found endemic in all maize-growing districts (Adams et al., 2014). The disease was officially reported in the Democratic Republic of Congo (DRC) predominantly in the western provinces of the north and south Kivu in 2014 (Lukanda et al., 2014). In Ethiopia, maize plants with MLN symptoms were first observed in 2014 prompting surveillance efforts which led to the first report (Mahuku et al., 2015b). There are reports of MLN in Southern Sudan (Mahuku et al., 2015a,b, unpublished results) and Burundi (Ministry of Agriculture surveillance reports, 2017).

Insect transmission of MLN causing viruses has been documented in Kenya (Mahuku et al., 2015a and Nyasani and Sevgan (unpublished results), 2012. Chrysomelid beetles have been demonstrated to transmit MCMV (Nault et al., 1978). The seed transmission rate of MCMV in maize has been documented to be very low, 0.04 % in the USA (Jensen et al., 1991), but MCMV seed transmission rates are not well documented in Kenya and eastern Africa.

Previous MLN distribution studies conducted in Kenya focused on a few counties where MLN viral diversity and other viral metagenomics studies were conducted (Mahuku et al., 2015a; and Wamaitha et al., 2018).

This study analyses the geographic distribution of MLN, its causative viruses, incidence and MLN symptom severity in 28 important maize growing counties covering all the maize growing Agro-ecological zones in Kenya. The study also highlights the genetic diversity of the viruses that cause MLN and examines recombination and its contribution to the evolutionary forces shaping the population of these viruses.

## 2 Materials and methods

### 2.1 Field survey and Data Analysis

A survey was carried out in 118 farms in 28 counties during the maize growing seasons in Kenya in 2015 and 2016. A questionnaire was administered to capture the survey data in the field. The counties surveyed were Kajiado, Bomet, Narok, Baringo, Nakuru, Marakwet, Nandi, Trans Nzoia, West Pokot, Uasin Gishu, Bungoma, Busia, Kakamega, Siaya, Migori, Homa Bay, Kisii, Kiambu, Kirinyaga, Embu, Meru, Machakos, Kitui, Makueni, Taita Taveta, Kwale, Kilifi and Tana River. Disease incidence and disease prevalence were determined by the percentage of the plants showing MLN symptoms in individual farms and the percentage of farms in a county with MLN symptoms, respectively. The recorded figures for incidence and symptom severity were the means from the farms visited in each county. A disease severity score was recorded using the 1−5 MLN symptom severity scale (CIMMYT Periodic Newsletter, June 2013). Fields having a maize crop as a pure stand or intercropped with other crops were selected and surveyed along designated routes in each county.

Seed fields were also surveyed in 11 counties under this study (Baringo, Nakuru, Taita Taveta, Tana River, Trans Nzoia, Elgeyo Marakwet, Machakos, Makueni, Meru, Embu and Uasin Gishu). The sampling protocol was the same as for farmers’ fields outlined.

Filled questionnaires were keyed into excel sheets and analyzed to provide information regarding disease incidence, prevalence and severity scores. Analysis of variance (ANOVA) and Tukey HSD of transformed data (arcsine transformation of proportion) was used to determine the significant difference and hierarchy of the reported data. Evaluation for varietal susceptibility to MLN was not done for all the popular commercial varieties/hybrids are susceptible to MLN (Semagn et al., 2015).

### 2.2 Sampling protocol

The staggered “X” pattern, recommended for most maize fields’ inspection and surveys (CDFA Phytosanitary Certification Manual, 1985), was adopted in the 118 sampled farms. Maize plants were examined for MLN symptoms along one side of the field then diagonally in a staggered pattern across rows to the far maize plants, and across the far side of the field and diagonally back to starting point. Young symptomatic leaves from five plants were sampled along the transect in each farm. Plants with MLN symptoms spotted away from the chosen pattern were also included in the five samples collected in each farmer’s field. The five samples from each farm were pooled together to make a composite sample representing that farm. In total, 118 maize leaf samples were collected from the field during the survey. This field inspection pattern ensured that all parts of the farm were adequately and proportionately represented in the plants inspected and sampled. This procedure was also used for the sampling of leaves from infected plants in seed fields. Three seed fields were visited in each of the 11 counties surveyed. A total of 33 composite samples were collected from these seed fields.

The samples were labeled, put in khaki bags, placed in a cool box containing dry ice, and transported to the laboratory for storage at −80 °C pending further laboratory analysis.

### 2.3 Ribonucleic acid extraction and quality check

RNA was extracted from the 118 samples and 33 samples from seed fields using the ZR RNA MiniPrep™ kit (Zymo Research Corporation, Irvine, CA) following the manufacturer’s recommendations. The overall quality of RNA was assessed by denaturing agarose gel pre-stained with GelRed™ staining dye (Biotium, Inc. Fremont, CA). The characteristic RNA 18S rRNA and 28S rRNA bands on the RNA gels were evaluated to ascertain the RNA integrity. The selected RNA samples for library preparation were treated with RNase-free DNase I (New England Biolabs inc., UK). The quality of the total RNA for library preparation was evaluated on the 2100 Bioanalyzer TapeStation system (Agilent Technologies, Inc., CA, USA). The RNA concentration was checked and determined on the Qubit® 2.0 Fluorimeter (Thermo Fisher Scientific, Wilmington, DE).

### 2.4 RT-PCR and DAS-ELISA

cDNA was prepared from 1 μg of RNA using a Maxima First Strand cDNA Synthesis kit (Thermo Fisher Scientific, Wilmington, DE) as per the instruction manual. Reverse transcription-polymerase chain reaction (RT-PCR) was conducted as described by Chen and colleagues (Chen et al., 2005). A two-step RT-PCR for MCMV was done on all the samples from the farmers’ fields and the seed fields to ascertain the presence of MCMV. MCMV-specific primers used were MCMV F 5′ −CCG GTC TAC CCG AGG TAG AAA – 3′ and MCMV R 5′ – TGG CTC GAA TAG CTC TGG ATT T – 3′. The 195 bp MCMV RT-PCR amplicons were analyzed by electrophoresis on a 1% agarose gel and visualized under UV light.

SCMV was detected by DAS-ELISA since the detection by RT-PCR with already existing published SCMV-specific primers (Alegria et al., 2003) did not work. DAS-ELISA for the detection of SCMV was done on the 118 samples from the farmers’ fields and the 33 samples from the seed fields. The commercial SCMV antiserum (Agdia, Elkhart, IN, USA) was used in a double antibody sandwich (DAS)-ELISA according to the manufacturer's instructions.

### 2.5 Illumina miseq sequencing

The TruSeq® Stranded Ribo-Zero RNA Sample Preparation Kit (Illumina, San Diego, USA) was used to prepare the libraries as per the manufacturer’s guidelines. Total RNA (500 ng) was used as starting materials for each sample in the protocol. The 48 samples were selected based on the geographical regions of the country surveyed for the analysis to reflect the national situation of MLN. Another important consideration was the RNA quality which determined the quality of the libraries generated from these samples for NGS analysis on the Miseq Illumina platform.

The samples tested positive for MCMV by RT-PCR and positive for SCMV by DAS ELISA but two negative samples each for MCMV and SCMV were also included.

The total RNA for each sample was converted into a library of template molecules for subsequent cDNA synthesis, cluster generation and eventually sequencing. The library concentrations generated for the 48 samples were checked using the Qubit® High Sensitivity D1000 Kit (Thermo Fisher Scientific, Wilmington, DE, USA) and sizing of individual libraries was done using the Agilent High Sensitivity D1000 ScreenTape System (Agilent Technologies, Santa Clara, CA, USA). Sequencing was done on the Illumina MiSeq platform (Illumina, San Diego, USA) at the BecA-ILRI Hub, Nairobi, Kenya. The samples were sequenced in two 150 bp paired end cycle runs with each run having 24 samples.

### 2.6 Sequence data analysis

Quality control of the data was performed using Fastqc (Andrews, 2010). Low-quality bases and adapters were trimmed off using Trimmomatic V 0.33 (Anthony et al., 2014). *De novo* assembly was performed on the remaining reads using metaSPAdes V 3.10. (Sergey et al., 2017). The resulting contigs were analyzed by BLASTN and TBLASTX (Zang et al., 200; Johnson et al., 2008) against a local download of NCBI nucleotide plant virus database and visualized using Krona (Ondov et al., 2011). The resultant contigs were also further analyzed by BLASTN to determine the sequence identity and similarity. Reference mapping of the assembled contigs to the most similar viral genomes was performed using CLC Genomics Workbench 5.5.1 (https://www.qiagenbioinformatics.com/). The resulting contigs were used in the phylogenetic analysis in MEGA V6 (Tamura et al., 2013).

### 2.7 Viral recombination analysis

The recombination detection program RDP4 v4.84 (Martin et al., 2015) was used to analyze both MCMV and SCMV sequences from this study for recombination events. The program RDP4 detects recombination breakpoints accurately and presents a friendly graphical interface for assessing various attributes in a recombination analysis process. The program simultaneously uses a range of different recombination detection methods to detect recombination events within aligned sequences. These methods include the BOOTSCANNING method (Salminen et al., 1995; Martin et al., 2005b), the GENECONV method (Padidam et al., 1995), the Maximum Chi-Square method (MAXCHI) (Maynard Smith, 1992; Posada and Crandall, 2001), the CHIMAERA method (Posada and Crandall, 2001), the Sister Scanning method SISCAN (Gibbs et al., 2000), the 3SEQ method (Boni et al., 2007), the VisRD method (Lemey et al., 2009) and theRDP4 BURT method. Only recombination events identified by at least four methods were further evaluated for the possibility of recombination.

The SCMV and MCMV sequence alignment files were used as input in RDP4. The default settings used by all the detection methods were utilized to complete automated analysis. There were two major phases in the automated analysis: the first involved the detection of recombination signals in the alignment and the second involved inference of the number and characteristics of unique recombination events that had been generated by the detected signals. The output of these analyses included the recombination region and breakpoint matrices, recombination positions and the analysis of the recombinant sequences detected with their deduced minor and major parents. The recombinant sequences were checked for the threshold parameters and only those that were analyzed by at least five implemented methods and those that had the required with p-values of <1.0 × 10^−6^ were accepted and considered to be a positive recombination event (Martin et al., 2005). Several analysis outputs were generated and recorded for further analysis and interpretation.

## 3 Results

### 3.1 Distribution, incidences, severity and prevalence of MLN

**Fig. 1:**
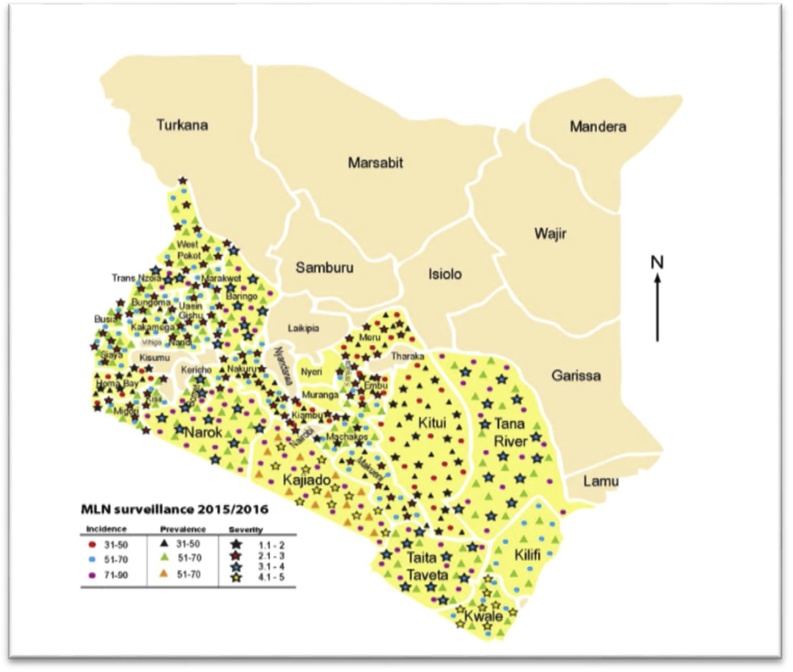
MLN incidence, prevalence and severity in the counties surveyed in 2015/2016.

Maize lethal necrosis disease was reported in the major maize growing regions of Kenya (Fig. 1). Counties in the south of the Great Rift Valley region (Kajiado, Bomet, Narok and Baringo) recorded the highest incidences of MLN as documented in Table 1. Kajiado county registered the highest MLN incidence countrywide with an incidence score of 68 % while Embu county in the Eastern region recorded the lowest disease incidence of 38 %. Coastal counties (Taita Taveta, Kwale, Kilifi and Tana River) had moderate MLN incidences (48 %–59 %).

**Table 1:** Incidences of MLN by counties in farmers’ fields for 2015/2016. Data which was in proportions (percentages) was arcsine transformed to meet model assumptions. The difference in the mean level of incidence among the different counties was statistically significant at α = 0.05 (F-Value = 8.654, P-value = 0.00000000000312).

County	Mean	Std error	Confidence interval	groups
Kajiado	67.88	2.34	67.88 ± 2.34	a
Bomet	63.64	2.04	63.64 ± 2.04	ab
Baringo	60.88	2.78	60.88 ± 2.78	abc
Narok	59.75	2.57	59.75 ± 2.57	abc
Trans-Nzoia	59.59	2.89	59.59 ± 2.89	abcd
Taita -Taveta	58.92	1.8	58.92 ± 1.8	abcde
West Pokot	68.32	2.12	68.32 ± 2.12	abcde
Bungoma	56.24	1.97	56.24 ± 1.97	abcdef
Tana River	55.68	1.32	55.68 ± 1.32	abcdef
Kilifi	54.93	1.22	54.93 ± 1.22	abcdefg
Uasin Gishu	53.11	0.6	53.11 ± 0.60	abcdefg
Kisii	52.55	2.13	52.55 ± 2.13	abcdefg
Marakwet	48.6	0.61	48.60 ± 0.61	abcdefg
Busia	57.21	1.72	57.21 ± 1.72	abcdefg
Migori	51.63	3.2	51.63 ± 3.20	bcdefg
Nandi	51.04	8.93	51.04 ± 8.93	bcdefg
Kirinyaga	50.95	2.2	50.95 ± 2.2	bcdefg
Makueni	48.93	1.98	48.93 ± 1.98	cdefg
Kwale	48.72	0.87	48.72 ± 0.87	cdefg
Machakos	48.72	0.5	48.72 ± 0.5	cdefg
Siaya	47.91	5.8	47.91 ± 5.8	cdefg
Kakamega	45.98	3.86	45.98 ± 3.86	cdefg
Nakuru	44.77	3.25	44.77 ± 3.25	defg
Meru	42.26	3.69	42.26 ± 3.69	efg
Kitui	41.86	2.85	41.86 ± 2.85	efg
Kiambu	41.5	2.63	41.5 ± 2.63	fg
Homa Bay	38.8	1.73	38.8 ± 1.73	g
Embu	37.88	3.95	37.88 ± 3.95	g

Bomet county registered the highest disease symptom severity of 3.6 while Homa Bay registered the lowest (1.6) on the 1−5 MLN disease symptom severity scale (Table 2). The counties in coast region namely Taita Taveta, Kwale and Kilifi had moderate symptom severity between 2 and 2.8. Similar results were observed in counties in the regions of Western Kenya (Kakamega, Bungoma, Busia, Siaya and Migori).

**Table 2:** MLN severity analysis. The difference in the mean level of severity among the different counties was statistically significant at α = 0.05 (F-Value = 5.271, P-value = 0.0000000592).

County	Mean Severity	Std error	Confidence interval	Groups
Bomet	3.56	0.55	3.56 ± 0.55	a
Kajiado	3.24	0.93	3.24 ± 0.93	ab
Baringo	3.23	0.39	3.23 ± 0.39	abc
Taita -Taveta	3.1	0.28	3.10 ± 0.28	abcd
Trans-Nzoia	3.08	0.59	3.08 ± 0.59	abcde
West Pokot	3.06	0.45	3.06 ± 0.45	abcde
Tana River	3.05	0.78	3.05 ± 0.78	abcde
Kilifi	2.98	0.54	2.98 ± 0.54	abcde
Narok	2.94	0.46	2.94 ± 0.46	abcde
Kwale	2.8	0.14	2.80 ± 0.14	abcde
Bungoma	2.73	0.46	2.73 ± 0.46	abcde
Nandi	2.65	0.35	2.65 ± 0.35	abcde
Migori	2.53	0.75	2.53 ± 0.75	abcde
Kisii	2.43	0.32	2.43 ± 0.32	abcde
Marakwet	2.62	0.33	2.62 ± 0.33	abcde
Busia	2.91	0.43	2.91 ± 0.43	abcde
Kakamega	2.37	0.55	2.37 ± 0.55	abcde
Uasin Gishu	2.35	0.21	2.35 ± 0.21	abcde
Kirinyaga	2.28	0.41	2.28 ± 0.41	bcde
Siaya	2.2	0.57	2.20 ± 0.57	bcde
Embu	2.1	0.36	2.10 ± 0.36	bcde
Nakuru	2.05	0.42	2.05 ± 0.42	cde
Makueni	2.02	0.27	2.02 ± 0.27	cde
Kiambu	2	0.17	2.00 ± 0.17	cde
Meru	1.93	0.32	1.93 ± 0.32	cde
Kitui	1.89	0.31	1.89 ± 0.31	de
Machakos	1.85	0.29	1.85 ± 0.29	de
Homa Bay	1.63	0.23	1.63 ± 0.23	e

The highest MLN incidence in seed fields was recorded in Baringo County and the neighboring Nakuru County where all the seed fields visited had MLN incidences of 100 % (Table 3). Baringo county also recorded the highest disease symptom severity of 3.5 followed by Nakuru and Taita-Taveta counties with symptom severity of 3.1 in seed fields. In general, seed fields in the counties of the north rift, south rift and the coast had higher disease incidence and symptom severity (Table 3). However, counties in the eastern part of the country recorded the lowest incidences and symptom severity in seed fields. These include Machakos, Makueni, Meru and Embu with an incidence range of 18%–45% and disease severity scores of 1.6–2.2 (Table 3).

**Table 3:** Incidence and severity of MLN in maize seed production fields in the counties surveyed.

County	Incidence Severity	
Baringo	100.0a	3.5a
Nakuru	100.0a	3.1b
Taita Taveta	75.0b	3.1b
Tana River	68.0c	2.8c
Trans Nzoia	63.0d	2.5d
Elgeyo-Marakwet	58.0e	2.3e
Machakos	45.0f	2.2ef
Makueni	35.0g	2.1f
Meru	25.0h	1.7gh
Embu	18.0i	1.6h
Uasin Gishu	16.0j	1.8g
Mean	54.8	2.42
LSD (P ≤ 0.05)	1.64	0.17
CV	1.8	4.3

Means in the same column are not significantly different at P ≤ 0.05.

### 3.2 RT-PCR and DAS-ELISA

**Fig. 2:**
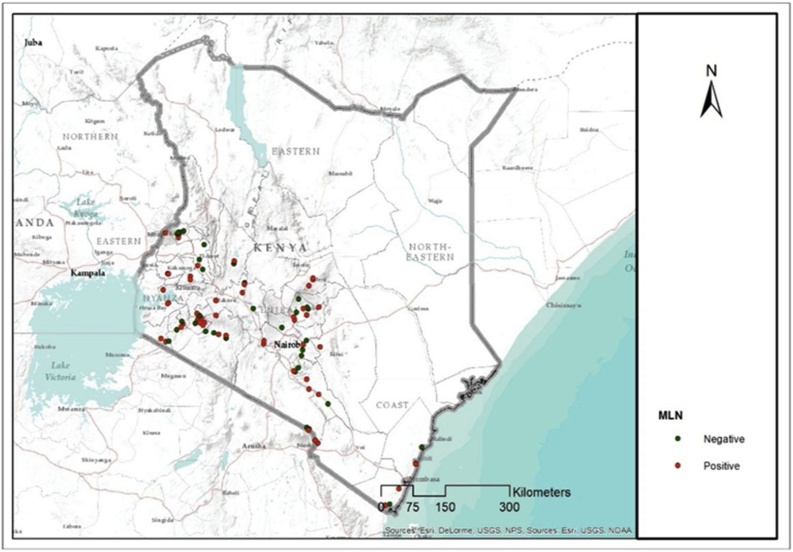
MLN (MCMV + SCMV) endemic areas among the sampled sites. The red points show the MLN (MCMV + SCMV) positive samples (by RT-PCR and DAS-ELISA, respectively) while the green points show samples negative for MLN viruses.

MCMV was detected by RT-PCR in 95 pooled leaf samples out of the 118 samples while SCMV was detected by DAS-ELISA in 85 samples out of the same 118 (Table 4 and Fig. 2). Seventy-three (73) samples showed a double infection of MCMV and SCMV). The high percentage of positive samples reflected the high number of symptomatic plants sampled. MCMV and SCMV were detected in all the 33 samples collected from the seed fields.

**Table 4:** MLN viruses testing results using RT-PCR for MCMV and DAS-ELISA for SCMV.

Diagnostics method	No. of Samples tested	Positive test (+ve)	% Positive
Farmers’ Fields			
MCMV (RT-PCR)	118	95	87%
SCMV (ELISA)	118	85	72%
MCMV + SCMV	118	73	62%
Seed Fields			
MCMV (RT-PCR)	33	33	100 %
SCMV (ELISA)	33	33	100 %

### 3.3 Viral genomes assembled from NGS sequencing data

The paired-end sequencing yielded 102,169,109 reads (35–151 bp) but 53,297,590 good quality reads were obtained (17–122 bp) after performing quality control. Following contigs assembly and BLASTN identification, most samples strongly showed the presence of MCMV and SCMV while a few samples showed the presence of Maize Yellow Mosaic virus (MaYMV), a recently described polerovirus (Adams et al., 2017 and Massawe et al., 2018). It was also demonstrated that there were no artefactual sequences that were generated in this study for the MCMV, SCMV and MaYMV had significant genome coverage by de novo and reference assemblies. No recombinant sequences were also detected in MCMV samples further indicating that artefacts were not introduced during the sequence assembly.

**Fig. 3:**
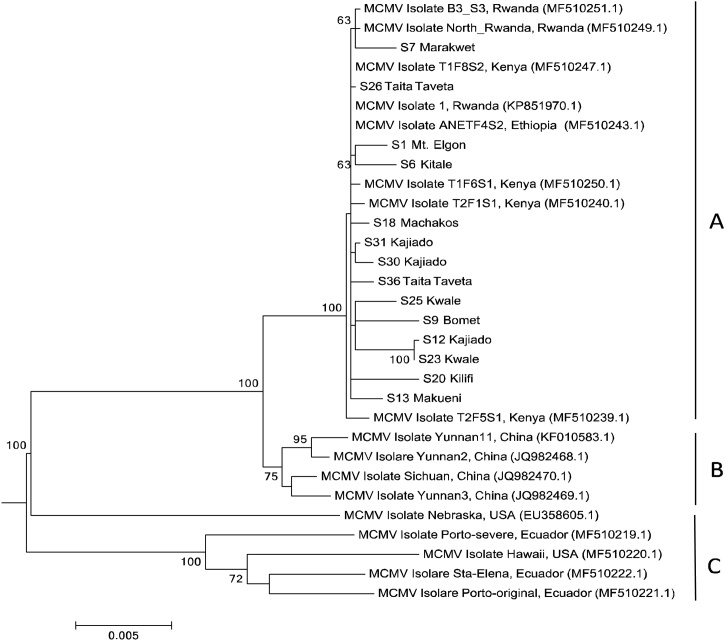
Phylogenetic analysis of the full genomes of 14 MCMV isolates recovered in this study with other full MCMV genome sequence accessions. The phylogeny was based on 4284 aligned nucleotide bases. The evolutionary history for MCMV viruses was inferred using MEGA version 6, Maximum Likelihood method based on the Tamura 2-parameter model at 1000 bootstraps (Tamura et al., 2013).

A total of 14 MCMV genomes were assembled in this study with genome lengths of 4403–4437 bp. Seven MCMV sequences were deposited in the NCBI nucleotide database (accession numbers: MH238449-MH238455). The isolate S6 with the accession number MH238454 was selected as a representative for further nucleotide and amino acid comparison with other MCMV isolates available in the NCBI nucleotide database. The Kenyan MCMV isolates identified in this study showed 99.75 % identity (Table 5) to previously reported isolates from Kenya, Ethiopia (Mahuku et al., 2015b) and Rwanda (Adams et al., 2014). The genomes assembled in this study were, however more divergent from those in Asia, Ecuador and the USA (Fig. 3). Table 5 shows the differences of the nucleotides and amino acids (aa) identified in the seven MCMV ORFs, namely P32, P50, P111, P31, P7a, P7b, and CP. The most diverse region was the ORF P31 showing 93.18 % identity with an isolate from Nebraska. However, MCMV isolates from these regions were similar in the P32, P11, and P7a ORFs. The CP region was the most similar with an aa identity range of 99.15–99.57 % while full genome sequences showed an overall similarity of 96.6–99.75%. The phylogeny of MCMV in Fig. 3 depicts 3 clades and shows that the samples in this study fall under clade A grouping with other Eastern Africa MCMV isolates. Clade B had isolates from China while clade C had isolates from the USA and Ecuador.

**Table 5:** Nucleotide and Amino acid sequence identities (%) between the study sample and isolates of MCMV from Kenya, Rwanda, China and USA.

Isolate	Accession	Country	Full genome (nt)	5′UTR (nt)	P32 (aa)	P50 (aa)	P111 (aa)	P7a (aa)	P7b (aa)	P31 (aa)	CP (aa)	3′UTR (nt)
MCMV_T1F6S1	MF510250	Kenya	99.75	100	100	99.54	99.79	100	100	99.64	99.57	100
MCMV_B3_S3	MF510251	Rwanda	99.75	100	99.65	99.54	99.48	100	100	99.28	99.57	100
MCMV_Yunnan2	JQ982468	China	99.02	99.09	98.26	98.85	99.48	100	100	98.2	99.57	99.68
MCMV_Nebraska	EU358605	Nebraska	96.64	97.27	97.23	96.34	97.82	98.52	96.87	93.18	99.15	97.8

**Fig. 4:**
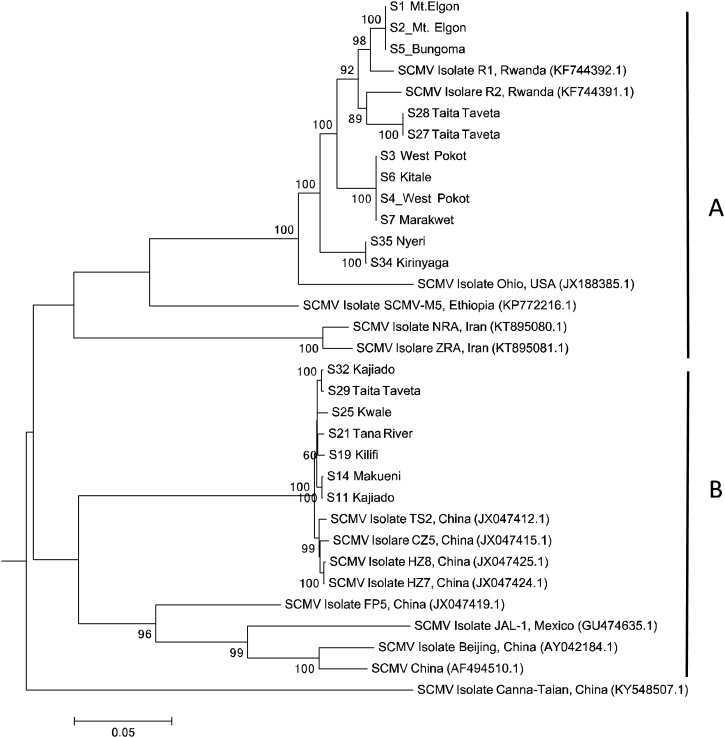
Phylogenetic analysis of the polyprotein gene of 18 SCMV isolates from this study, with other polyprotein SCMV accessions from NCBI. The phylogeny was based on 9099 aligned nucleotide bases. The evolutionary history was inferred with MEGA version 6, Maximum likelihood method based on the General Time Reversible model at 1000 bootstraps (Tamura et al., 2013).

In this study, 18 full genomes of SCMV with lengths ranging from 9440 to 9647 bp (all including the polyprotein and variable lengths in the 5′UTR and 3′UTR) were recovered together with 21 partial SCMV genome sequences (1500–6900 bp). The SCMV phylogenetic tree in Fig. 4 shows the presence of 2 clades: 11 SCMV genomes detected in this study clustered with SCMV isolates from Rwanda and Ohio, USA, Ethiopia and Iran (Clade A), while 7 SCMV genomes clustered with SCMV strains from China and Mexico (Clade B). The genome sequences of SCMV were quite diverse, the sequence identity of the polyprotein gene in our samples ranged from 89.8%–100%. Amino acids identity analysis for the SCMV polyprotein revealed that SCMV isolates from neighboring counties of West Pokot and Marakwet were 99 %–100 % identical in composition. Real time qPCR primers and probe were designed for these Kenyan SCMV isolates using the qPCR Primer Quest tool, Integrated DNA Technologies Inc.

(https://www.idtdna.com’primerquest). The primers target the 7375–7521 SCMV genomic region with an expected amplicon of 186 bp. These primers are F: 5′-AGCCGAAATCAGACCAATAGAG-3′, R: 5′-AAGCGATTCCAACCTCCATAG-3′ and the Probe: TCACACCATTTAGAAGGCCCATGGAC.

Partial sequences of MaYMV were generated by de novo and reference sequence assembly in this study. They were analyzed by BLASTN against a local Plant Virus Genome Database (PVGDB) (http://www.ncbi.nlm.nih.gov/genome/viruses). Our MaYMV sequences were highly similar to already documented MaYMV accessions in the NCBI nucleotide database. They were 99.4 % similar to MH205607 (Wamaitha et al., 2018) hereby described as MYDMV-RMV from Kenya and MF684367, MaYMV from Ethiopia. They were also 99 % similar to MF974579, a Kenyan isolate (Massawe et al., 2018) and MF425856 Ethiopian isolate (Adams et al., 2017).

### 3.4 MCMV single infection

There were 4 samples where only MCMV as an MLN causing virus was recovered. These were S9-Bomet, S12-Kajiado, S16-Machakos and S-18_Machakos (Table 6). These samples, however, posted high MLN symptom severity (3.0–3.5 on a 0–5 MLN severity scale) showing full development of MLN just like those samples with co-infection with SCMV. The specific samples tested negative for SCMV by DAS-ELISA, NGS, and other potential potyviruses and poleroviruses, especially MaYMV, detected in this study were absent. No SCMV sequences were generated from these samples and the BLASTN results showed only the presence of MCMV and Maize Yellow dwarf virus (Table 7). The Krona BLASTN display for these four samples also showed only MCMV presence while all other samples with MLN showed the presence of both MCMV and SCMV.

**Table 6:** Samples that showed only MCMV infection both through NGS and laboratory testing for MCMV and SCMV and their corresponding MLN symptom severity.

Sample ID	Contig Length	Symptom severity (1−5)
S9_MCMV_Bomet	4405	3.5
S12_ MCMV_Kajiado	4403	3.2
S16_MCMV_Machakos	4404	2.1
S18_ MCMV_Machakos	4403	1.9

**Table 7:** Summary of the BLAST results of assembled contigs. The plant viruses detected are; MCMV, SCMV, Maize Yellow Mosaic Virus (MaYMV). The percentage similarity, genome coverage and the accessions of the blasted sequences are illustrated. Isolates S9, S12, S16 and S18 with the Asterix sign (*) have only MCMV infection without SCMV or MaYMV.

Sample ID	Virus	Strain	% Similarity	E-Value	Coverage	Accessions
S1	MCMV	TF1F6S1	99	0	25158.9	MF510250.1
S1	SCMV	R1	99	0	59.8	KF744392.1
S2	MCMV	Isolate 1	99	0	44554.5	KP851970.3
S2	SCMV	R1	98	0	153.85	KF744392.1
S3	MCMV	TF1F6S1	99	0	31,978	MF510250.1
S3	SCMV	R1	99	0	82	KF744392.1
S4	MCMV	TF1F6S1	99	0	9766.32	MF510250.1
S4	SCMV	R1	99	0	31.9	KF744392.1
S5	MCMV	TF1F6S1	99	0	14678.4	MF510250.1
S5	SCMV	R1	98	0	31.6	KF744392.1
S6	MCMV	Isolate 1	99	0	16979.2	KP851970.3
S6	SCMV	R1	95	0	36.7	KF744392.1
S7	MCMV	Isolate B3_S3	99	0	14705.9	MF510251.1
S7	SCMV	R1	95	0	36.28	KF744392.1
S9*	MCMV	Isolate 1	99	0	11220.3	KP851970.3
S10	MCMV	Isolate B3_S3	99	0	5043.9	MF510251.1
S10	SCMV	HZ8	99	0	12.25	JX047392.1
S11	MCMV	Isolate B3_S3	99	0	9440.7	MF510251.1
S11	SCMV	HZ8	99	0	22.14	JX047424.1
S12*	MCMV	Isolate 1	99	0	1763.4	KP851970.3
S13	MCMV	Isolate 1	99	0	956.56	KP851970.3
S13	SCMV	SCMV-M5	97	0	4.1	KP772216.1
S14	MCMV	Isolate B3_S3	99	0	5684.63	MF510251.1
S14	SCMV	HZ8	99	0	20.27	JX047424.1
S15	MCMV	Isolate 1	99	0	4302.56	KP851970.3
S15	SCMV	CD1	99	0	15.98	JX047392.1
S16*	MCMV	TF1F6S1	99	0	9230.35	MF510250.1
S18*	MCMV	Isolate 1	99	0	271.96	KP851970.3
S19	SCMV	HZ7	99	0	19.79	JX047424.1
S20	MCMV	Isolate 1	99	0	3223.81	KP851970.3
S20	SCMV	R1	99	0	4.8	KF744392.1
S21	SCMV	HZ7	99	0	70.5	JX047424.1
S22	MCMV	TF1F6S1	99	0	6.8	MF510250.1
S23	MCMV	Isolate 1	99	0	281.82	KP851970.3
S24	MCMV	Isolate B3_S3	99	0	332.1	MF510251.1
S25	MCMV	Isolate B3	99	0	6651.55	MF510251.1
S25	SCMV	HZ7	99	0	535.2	JX047424.1
S25	MaYMV	T2F3S4	99	0	3.8	MF425876.1
S25	MaYMV	T2F3S4	99	0	5.1	MF425876.1
S25	MaYMV	T2F3S4	99	0	2.2	MF425876.1
S25	MaYMV	T2F3S4	99	0	4.8	MF425876.1
S25	MaYMV	T2F3S4	99	0	2.2	MF425876.1
S26	MCMV	T1F6S3	99	0	10538.1	MF510244.1
S26	MaYMV	T2F3S4	99	0	1.2	MF425876.1
S26	MaYMV	T2F5S2	99	0	2.6	MF425878.1
S26	SCMV	HZ7	99	0	68.8	JX047424.1
S26	SCMV		98	0	12.3	KF744391.1
S27	MCMV	Isolate 1	99	0	31241.1	KP851970.3
S27	MaYMV	T2F5S1	99	0	1.6	MF425877.1
S27	MaYMV	T2F5S1	99	0	3.5	MF425877.1
S27	MaYMV	T2F3S4	99	0	1.6	MF425874.1
S27	SCMV	R2	96	0	141.1	KF744391.1
S28	MCMV	Isolate B3	99	0	12466.4	MF510251.1
S28	MaYMV	T2F5S1	99	0	1.5	MF425877.1
S28	SCMV	R2	96	0	91	KF744391.1
S29	MCMV	Isolate B3	99	0	5008.95	MF510251.1
S29	SCMV	HZ7	99	0	186.164	JX047424.1
S30	MCMV	Isolate 1	99	0	6724.5	KP851970.3
S31	MCMV	Isolate 1	99	0	11782.5	KP851970.3
S32	MCMV	Isolate B3	99	0	8510.1	MF510251.1
S32	MaYMV	T2F5S2	99	0	1.1	MF425878.1
S32	SCMV	HZ7	99	0	131.1	JX047424.1
S33	MCMV	T1F6S3	99	0	11680.6	MF510244.1
S33	MaYMV	T1F8S2	99	4e-154	1.35	MF425872.1
S33	SCMV	R2	96	0	63.9	KF744391.1
S34	MCMV	T1F6S3	99	0	11897.4	MF510244.1
S34	MaYMV	T2F5S2	99	0	1.9	MF425878.1
S34	SCMV	R2	95	0	111.9	KF744391.1
S34	MCMV	T1F6S1	99	0	5709	MF510250.1
S35	SCMV	R2	95	0	74.5	KF744391.1
S36	MCMV	Isolate 1	99	0	705.2	KP851970.3

### 3.5 Viral recombination analysis for MCMV and SCMV

Potential recombination events were detected in 11 out of 18 SCMV genome sequences but MCMV sequences recovered in this study did not generate any recombinants. However, only 2 SCMV genome sequences were considered to be recombinants with different possible major and minor parents by at least four different RDP4-implemented methods with acceptable P values of <1.0 × 10^−^°^6^ (Table 8). The recombinant SCMV isolates detected were S2 Bungoma and S35 Nyeri (Table 8).

**Table 8:** Detected recombination events of several SCMV isolates by at least 5 recombination evaluation methods. The two from the study samples were S2 Bungoma and S35 Nyeri. The corresponding P values and the recombination sites are illustrated. The methods key; R-RDP, G-GENECOV, B-BootScan, M-MaxiChi, C-Chimaera and S-Siscan were the recombination analysis methods used.

Recombinant	Programs supporting recombination	Major Parent	Minor Parent	P-Value	Recombination sites
S2 Bungoma	RGBMCS	KF744392	S7 Marakwet	2.804 × 10^−50^	4619–9567
MG 932079.1	RGBMCS	KP860935	S7 Marakwet	6.423 × 10^−37^	5532 – 7977
MF467403.1	RGBMCS	MF467404.1	S2 Bungoma	1.359 × 10^−16^	4063 – 4137
MF467403.1	RGBMC	S32 Kajiado	S2 Bungoma	7.728. × 10^−16^	4598 – 9443
JX047419	RBGMCS	S35 Nyeri	MG 932079.1	1.678 × 10^−26^	7281 – 8948
MG932076.1	RGMCS	S27 Taveta	KF 744392.1	4.380 × 10^−10^	8078 – 9562
S35 Nyeri	RGBMCS	S27 Taveta	S7 Marakwet	6.138 × 10^−15^	4653 – 9562
MG930076.1	RGBMC	S27 Taveta	S32 Kajiado	9.607 × 10^−141^	8115 – 9571

In the S2 Bungoma recombinant sequence, the major parent was KF744392, an SCMV isolate from Rwanda while the minor parent was S7 Marakwet isolate. S35 Nyeri had S27 Taita Taveta as a major parent and S7 Marakwet as the minor parent.

**Fig. 5:**
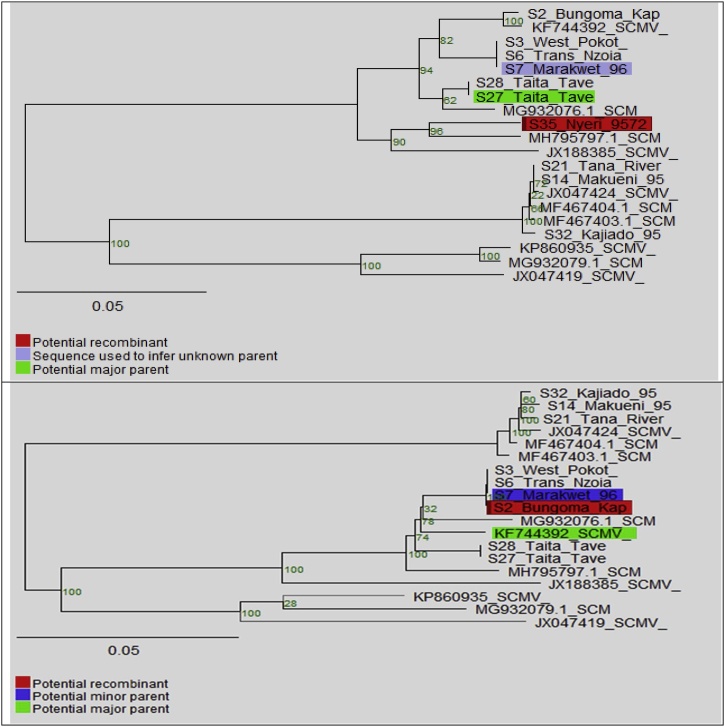
Neighbor joining trees generated after accepting S2 Bungoma and S35 Nyeri as recombinants with several recombination events detected. Sample S7 Marakwet and KF744392 are the minor and major parents for the recombinant S2 Bungoma, respectively. The Neighbor joining trees were based on the transition transversion method model at 100 bootstraps (Kimura, 1980).

The Neighbor joining trees illustrated in Fig. 5 shows sample S2 Bungoma and S35 Nyeri as recombinants sequences. Isolate S7 Marakwet was used to infer unknown minor parent and S27 Taita Taveta was identified as the major parent for the S35 Nyeri recombinant sequence.

It was also observed that some SCMV accessions in the GenBank had some of our isolates as minor parents e.g. MG 932079.1, a Kenyan isolate from Kirinyaga county having KP880903, an Ethiopian isolate, as the major parent and S7 Marakwet, as a minor parent. The same was observed for MF467403.1 (Tanzania) which had MF467404.1 (Tanzania) as a major parent and S2 Bungoma as a minor parent. As such, several other SCMV accessions had our sequences either as minor or major parents. There were also weak recombinant signals showing Nigeria and USA isolates as minor parents of our isolates.

**Fig. 6:**
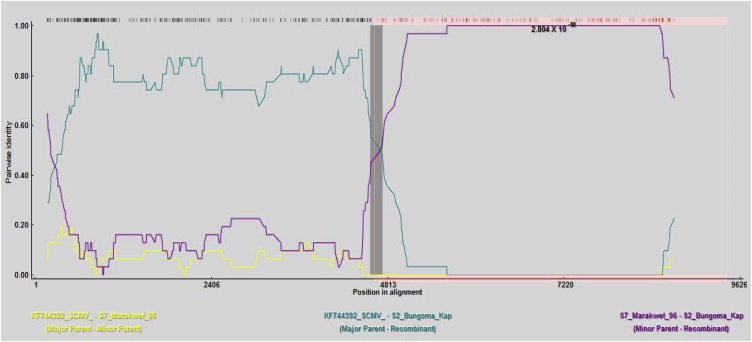
Pairwise identity of the sample sequences showing the recombinant S2 Bungoma SCMV sequence and the possible parents of this recombinant sequence: KF744392 identified as the major recombinant and S7 Marakwet as a minor parent. The recombination event was detected in the 4619 – 9567 bp region of the genome.

Recombination analysis through RDP4 also allows elucidating the exact points along the viral genome where genetic recombination has occurred, as illustrated in Fig. 6. The recombination event occurred along position 4619–9567 on the SCMV genome for sample S2 Bungoma having KF744392 and S7 Marakwet as major and minor parents, respectively. The size of the fragments from both the major and minor parents can be determined in each recombinant sequence.

## 4 Discussion

This study presents an update on the occurrence, distribution and genetic diversity of MLN-causing viruses in Kenya and gives a pioneer report on the viral recombination profiles for SCMV. MCMV and SCMV were prevalent in the country; however, MCMV was more prevalent than SCMV in all the 28 counties surveyed. MaYMV was also detected in some samples. MCMV and SCMV were present in all the seed fields surveyed. This may be due to the natural occurrence of insect vectors (aphids, thrips and beetles) and abundant sources of inoculum because of the continuous cropping in seed production fields. It has been demonstrated that the prevalence of SCMV is dependent on many factors but the sources of inoculum through vector populations can influence the periodicity of SCMV development and establishment (Louie, 1980).

The counties in the South Rift region had the highest incidences and symptom severity of MLN (Table 1, Table 2). It was evident that MLN was severe in the regions where there is continuous maize growing especially in counties with supplementary irrigation like Baringo, Kajiado and Taita Taveta compared to areas with one or two distinct maize growing seasons. Higher incidence and severity of the disease symptoms in seed fields were recorded in Baringo, Taita Taveta and Tana River counties (Table 3). Many seed companies contract farmers in irrigation schemes in these counties for seed production leading to accumulation of the viral inoculum to the ever-present host maize plants

The low to relatively moderate incidence and prevalence of MLN in counties of the north rift region like Trans Nzoia, West Pokot, Marakwet and Uasin Gishu and counties in western Kenya was partly related to the existence of one major rain-fed maize cropping season. Farmers in these counties also have a higher certified seeds’ adoption rate compared to farmers from other counties due to higher levels of awareness and proximity to local seed sellers (Ouma et al., 2014). As such, these farmers might be using seeds with low levels of infection and contamination by MLN causing viruses. The low MCMV levels in certified seeds may also be attributed to the recently implemented strict MLN seed certification regulations by the Kenya Plant Health Inspectorate Service (KEPHIS), the official seed certification agency in Kenya for MLN viruses-free seed production (www.kephis.org).

Counties in the eastern part of the country recorded the lowest incidence and symptom severity. (Table 1, Table 2). This is attributed to one maize growing season while pulses (mainly beans and green grams) are grown in short season with unreliable rainfall. This helps to lower the viral inoculum in the fields that breaks the vector lifecycles that leads to low levels of MLN infections (https://www.cabi.org/isc/datasheet/119,663).

Monitoring MLN incidence in seed fields is very important for seed lots infected by these viruses may fuel the spread of MLN. The rate of transmission of MCMV through seed is low as reported in previous studies (Jensen et al., 1991). However, the presence of MCMV in commercial seed lots may lead to amplified spread by insect vectors endemic in the maize growing areas. The high MLN incidence in Trans Nzoia, Uasin Gishu and Baringo counties with many seed production fields may result in many seedlots infected by MCMV. Precautionary measures need to be put in place to limit seed infection and transmission.

Molecular analysis of the samples through NGS showed that SCMV was the only potyvirus identified in this study that coinfects maize together with MCMV thereby causing MLN. However, MaYMV, a polerovirus was also found in a few samples. Coinfection of MCMV and SCMV viruses in farmers’ fields was recorded in 73 samples (Table 4) evenly distributed across the country (Fig. 1, Fig. 2). Moderate to high MLN severity scores were also recorded in these 73 samples during the survey. In the United States, it was found that when maize plants are co-infected with MCMV and one of several potyviruses including MDMV, SCMV or the tritimovirus WSMV, leaves and stems of infected plants developed severe systemic necrosis known as corn lethal necrosis (CLN) disease which is an MLN synonym (Scheets, 1998). The same scenario was observed in this study where samples with both MCMV and SCMV exhibited severe MLN symptoms. Studies on coinfection of MCMV and SCMV demonstrated increased accumulation of MCMV and virus-derived small interfering RNAs (vsiRNAs) from MCMV (Xia et al. 2016). This indicates increased RNA silencing activity by the plant’s defense mechanism against the virus infection. In one such study, it was demonstrated that the helper component protease (HC-Pro) encoded by potyviruses mediates suppression of post transcriptional gene silencing (PTGS) enhancing the pathogenicity and accumulation of other heterologous viruses (Pruss et al., 1997; Gonza´lez-Jara et al., 2005; Syller, 2012). Though they belong to the same family (*potyviridae*), WSMV HC-Pro has been shown not to influence disease synergism with MCMV (Stenger et al., 2007). Instead, WSMV mediates synergistic interactions with other viruses by utilizing a gene other than HC-Pro for PTGS suppression.

Investigation on ultrastructural damage on chloroplasts in bundle sheath cells of maize leaves infected by both MCMV and SCMV had much smaller starch grains in the chloroplasts (Wang et al., 2017) which indicates that co-infection leads to the severity of the disease. It has also been demonstrated that there is an increase in the MCMV virus titer in mixed infections with *Maize dwarf mosaic virus* (MDMV), strain-B (Goldberg and Brakke, 1987) or with *Johnsongrass mosaic virus* (JGMV), a potyvirus that has been recently reported to co-infect maize with MCMV causing MLN (Stewart et al., 2017); however, these viruses were not detected in this study.

MaYMV was detected in 5 samples in this study. MaYMV has been detected in maize samples from recent MLN survey studies in eastern Africa and other MLN endemic countries worldwide (Asiimwe et al., 2019). In eastern Africa, MaYMV has been found present in all recent MLN related studies though some publications have described it as Maize yellow dwarf mosaic virus (MYDMV) (Adams et al., 2017: Massawe et al., 2018: Wamaitha et al., 2018; Read et al., 2019; Asiimwe et al., 2019; Kiruwa et al., 2019: Stewart et al., 2020). The recent study of maize infecting viruses in South Korea (Lim et al., 2018) identified MaYMV prevalence but no link to MLN related symptoms. Though MaYMV is frequently found in samples with MLN causing viruses, there has been no direct link to it contributing to the MLN disease complex. Polerovirus, just like potyviruses, are known to suppress post transcriptional gene silencing but it is not yet clear if this factor excerbarates MLN proliferation (Baumberger et al., 2007).

However, there were some samples in this study that were SCMV negative by DAS-ELISA and MCMV positive by RT-PCR but showed severe MLN symptoms. These isolates were S9-Bomet, S12-Kajiado, S6-Machakos and S18-Machakos. These samples posted moderate to high MLN symptom severity scores (1.9–3.5 on a 1−5 MLN severity scale) showing full development of MLN just like those with co-infected with SCMV (Table 6). Samples S9 and S12 had symptom severity scores of 3.5 and 3.2 respectively. This indicates that MCMV alone can lead to severe infections like those arising from coinfections. This observation was also supported by the NGS data where samples that had clear MLN symptoms showed infection by only MCMV through recovered MCMV sequences, BLASTN results (Table 6, Table 7).

MCMV genome sequence comparison with accessions from China, Africa and the USA reveals that MCMV is a highly conserved virus with identities ranging from 96 % to 99 %. This is typical of members of *Tombusviridae* where the diversity of nucleotides documented are between 0−0.02 with MCMV in the genus *Machlomovirus* recording lowest nucleotide diversities of 0.01 (Varanda et al., 2014 and Braidwood et al., 2018). Comparison of the amino acid sequences of the viral proteins also exhibited high similarities, especially in the P7a, P111 and the CP regions as indicated in Table 5. The 5′ and 3′UTRs were highly conserved in all the isolates under investigation confirming reports from previous studies (Mahuku et al., 2015a; Braidwood et al., 2018; Wamaitha et al., 2018).

Phylogenetic analysis suggested a potentially common origin for Eastern Africa and Asian MCMV isolates. The studied samples clustered in clade A (Fig. 6) together with MCMV isolates from Kenya, Ethiopia and Rwanda indicating a very close relationship of MCMV strains circulating in Eastern Africa. The closest neighbor (clade B) contains MCMV isolates from China suggesting that the MCMV strains endemic in Eastern Africa may have had its origin from China. The MCMV isolates characterized in this study seemed to be more divergent from the MCMV isolates in the USA and South America. Recent studies on global phylogeny of MCMV by Braidwood and colleagues (Braidwood et al., 2018) also showed a close similarity between the China and Eastern Africa isolates. However, this study reveals a more distinct strain in East Africa which has proved to be more virulent compared to the strains of the Americas and Asia (CIMMYT MLN Epidemiology research Project report, 2019). This is evident from the reported yield losses of 50–100 % in Kenya (Mahuku et al., 2015a; De Groote et al., 2016) and up to 90 % in Ethiopia (Girma et al., 2018). Initial reports alluded to MCMV not varying in its infection and pathogenicity (Wang et al., 2017) but the infection patterns in eastern Africa show a different scenario. Materials tolerant to MCMV in the US have been found to be susceptible to MLN in eastern Africa indicating differences in the pathogenicity of the strains in the US and those circulating in eastern Africa (CIMMYT periodic reports, 2018).

Phylogenetic analysis of SCMV recovered sequences revealed 2 distinct clusters of SCMV (Fig. 4). This analysis also showed some geographical clustering as seen for the isolates from Trans Nzoia, West Pokot and Marakwet counties which border each other in the north rift region of the country. The same trend was observed with SCMV isolates from counties of Kajiado, Taita Taveta, Kwale, and Tana River in the South rift and coast region. There were no other potyviruses recovered through NGS in this study, contrary to other studies where a potyvirus, *Johnsongrass mosaic virus* (JGMV) (Stewart et al., 2017; Wamaitha et al., 2018) was found to be present in MLN infected plants.

This study also evaluated the viral recombination of the identified viruses using the recombination detection program RDP4 v4.84 (Martin et al., 2015). Our Sample S2 Bungoma and S35 Nyeri gave strong recombination signals and were recombinants as explained earlier. The recombination events were also identified among SCMV isolates found in the eastern Africa region with an exception of one isolate from China (JX047419), which had our isolate S35 Nyeri and another Kenyan isolate MG932071 (Wamaitha et al., 2018) as a major and minor parent respectively. Generally, recombination signals were strong among eastern Africa isolates mostly from Kenya, Rwanda, Tanzania and Ethiopia. A typical case is a Tanzanian isolate, MF 467403.1 which had our sample S32 Kajiado as a major parent and S2 Bungoma as a minor parent. This is expected for Kajiado borders Tanzania and this isolate MF 467403.1 (Kiruwa et al., 2019) originates in Arusha, a district that borders Kenya.

Recombination signals were not detected in the MCMV genomes analyzed. This is partly because the virus is largely conserved with little genetic variation across the globe (Braidwood et al., 2018). As illustrated in the MCMV phylogenetic analysis, MCMV isolates are highly similar hence genetically conserved with minimal evidence of rapid evolution. There is a clear separation of MCMV isolates from different world regions, indicating that there has been no recombination between MCMV genomes in geographically isolated regions (Braidwood et al., 2018).

Viral genetic recombination is a natural phenomenon and has been demonstrated to play an important role in the evolution of viruses (King et al., 1982). Recombination in viruses has also been observed to be a pervasive process that generates diversity in most viruses (Valli et al., 2007 and Martin et al., 2015). It occurs when at least two viral genomes coinfect the same host cell and exchange genetic segments possibly creating new variants for viruses to adapt to new hosts and environments by selective pressures (Perez-Losada et al., 2015). Similar studies on SCMV diversity in Shanxi, China revealed that SCMV not only evolves by divergence from common ancestors but also by inter-viral recombination (Xie et al., 2016). A considerable number of *potyviruses* can be regarded as successful products of several recombination events (Goncalves et al., 2011). Intra-species recombination is important in *potyviridae* evolution (Li et al., 2013) as demonstrated in this study for the eastern Africa SCMV isolates.

The evolutionary pattern of SCMV needs to be continuously assessed in the country to determine if there are any virulent or more severe strains for which commensurate management strategies must be designed. Molecular diagnostic protocols need to be updated by incorporating new primer sequences designed by analyzing the new SCMV sequences generated and publicly available. It has been shown previously by KEPHIS (KEPHIS annual report, 2016) that primer sequences for SCMV from other sources do not work for the Kenya SCMV isolates. This was the case in this study where the SCMV primers used did not amplify the target Kenyan SCMV isolates hence the reason why DAS-ELISA was used in detecting SCMV. This confirms the high level of diversity in the SCMV sequences across the globe hence the need for specific primers for the local isolates. Real time qPCR primers were designed for these Kenyan SCMV isolates. The primers and probe targets the region 7375–7521 with an expected amplicon of 186 bp.

There is a need to implement mitigation strategies simultaneously to effectively combat this devastating maize disease based on proposed MLN management models (Hilker et al., 2017). There is an initiative to strengthen the National Plant Protection Organizations (NPPOs) capacity to test for MLN viruses, especially MCMV, in seed lots for seed certification and on seed and grain movement across borders. Adoption of MLN free seed production protocols developed by partners and seed companies in eastern African countries where MLN is endemic will reduce seedlots infected with MCMV (Prasanna et al., 2020). With the current extremely high levels of MCMV and SCMV infections in seed fields, this initiative is very valuable. There is also an initiative aiming at studying various factors affecting MLN epidemiology in eastern Africa (CIMMYT annual report, 2018). Several studies are being pursued to understand MCMV transmission through commercial seed in countries where the virus is endemic to facilitate more effective control (Annual MLN Epidemiology project report 2019). Highly important is generating knowledge about the relationship between seed infestation and seed transmission of MCMV, agronomic mitigation practices, crop rotations (especially with legumes), and prevention measures for the spread of MCMV from endemic to non-endemic areas. There is also a need for further studies to ascertain the sum effect of other viruses and abiotic factors that complicate the etiology of MLN in Kenya and by extension in eastern Africa.

## CRediT authorship contribution statement

**Francis M. Mwatuni:** Conceptualization, Methodology, Writing - original draft, Formal analysis. **Aggrey Bernard Nyende:** Funding acquisition, Supervision. **Joyce Njuguna:** Software, Data curation. **Xiong Zhonguo:** Supervision. **Eunice Machuka:** Methodology. **Francesca Stomeo:** Writing - review & editing, Resources, Supervision, Funding acquisition.

## Declaration of Competing Interest

None.

## References

[bib0005] Adams I.P., Miano D.W., Kinyua Z.M., Wangai A., Kimani E., Phiri N., Reeder R., Harju V., Glover R., Hany U., Souza-Richards R., Deb Nath P., Nixon T., Fox A., Barnes A., Smith J., Skelton A., Thwaites R., Mumford R., Boonham N. (2012). Use of next-generation sequencing for the identification and characterization of Maize chlorotic mottle virus and Sugarcane mosaic virus causing maize lethal necrosis in Kenya. Plant Pathol..

[bib0010] Adams I.P., Harju V.A., Hodges T., Hany U., Skelton A., Rai S., Deka M.K., Smith J., Fox A., Uzayisenga B., Ngaboyisonga C., Uwumukiza B., Rutikanga A., Rutherford M., Ricthis B., Phiri N., Boonham N. (2014). First report of maize lethal necrosis disease in Rwanda. New Dis. Rep..

[bib0015] Adams I., Braidwood L., Stomeo F., Phiri N., Uwumukiza B., Feyissa B., Mahuku G., Wangai A., Smith J., Mumford R. (2017). Characterising maize viruses associated with maize lethal necrosis symptoms in sub-Saharan Africa. bioRxiv..

[bib0020] Alegria O.M., Royer M., Bousalem M., Chatenet M., Peterschmitt M., Girard J.-C., Rott P. (2003). Genetic diversity in the coat protein coding region of eighty-six sugarcane mosaic virus isolates from eight countries, particularly from Cameroon and Congo. Arch. Virol..

[bib0025] Andrews S. (2010). FastQC: A Quality Control Tool for High Throughput Sequence Data. http://www.bioinformatics.babraham.ac.uk/projects/fastqc.

[bib0030] Anthony M.B., Marc L., Bjoern U. (2014). Trimmomatic: a flexible trimmer for Illumina sequence data. Bioinformatics.

[bib0035] Asiimwe T., Stewart L.R., Kristen W., Massawe D.P., Jovia K., Redinbaugh M.G. (2019). Maize lethal necrosis viruses and other maize viruses in Rwanda. Plant Pathol..

[bib0040] Baumberger N., Tsai C.-H., Lie M., Havecker E., Baulcombe David C. (2007). The polerovirus silencing suppressor P0 targets ARGONAUTE proteins for degradation. Curr. Biol..

[bib0045] Boni M.F., Posada D., Feldman M.W. (2007). An exact nonparametric method for inferring mosaic structure in sequence triplets. Genetics.

[bib0050] Braidwood L., Quito-Avila Diego F., Cabanas D., Bressan A., Wangai A., Baulcombe D.C. (2018). Maize chlorotic mottle virus exhibits low divergence between differentiated regional sub-populations. Sci. Rep..

[bib0055] Brandes E.U. (1920). Mosaic disease of corn. J. Agric. Res..

[bib0060] CDFA (1985). CDFA Phytosanitary Field Inspection Manual.

[bib0065] Chen K., Knorr C., Bornemann-Kolatzki K. (2005). Targeted oligonucleotide-mediated microsatellite identification (TOMMI) from large-insert library clones. BMC Genet..

[bib0070] CIMMYT (2012). Periodic Newsletter Reports.

[bib0075] CIMMYT (2013). Periodic Newsletter Reports.

[bib0080] CIMMYT (2018). CIMMYT Annual Report.

[bib0085] CIMMYT MLN (2018). Annual CIMMYT MLN Epidemiology Project Report.

[bib0090] CIMMYT MLN (2019). Epidemiology Research Project Report.

[bib0095] De Groote H., Oloo F., Tongruksawattana S., Biswanath D. (2016). Community-survey based assessment of the geographic distribution and impact of maize lethal necrosis (MLN) disease in Kenya. Crop. Prot..

[bib0100] FAO (2016). FAO Quarterly EAC Reports on Food Security.

[bib0105] FAOSTAT (2016). Food and Agriculture Organization of the United Nations. http://faostat.fao.org/site/339/default.aspx.

[bib0110] Gibbs M.J., Armstrong J.S., Gibbs A.J. (2000). Sister-scanning: a Monte Carlo procedure for assessing signals in recombinant sequences. Bioinformatics.

[bib0115] Girma D., Temesgen D., Messele H., Gezahegn B., Midekissa D., Temesgen C., Yitayih G., Dufera T., Mezigebu D., Tolera K., Girum A. (2018). Prevalence, distribution and impact of maize lethal necrosis disease (MLND) in Ethiopia. Pest Man. J. Eth..

[bib0120] Goldberg K.B., Brakke M.K. (1987). Concentration of Maize chlorotic mottle virus increased in mixed infections with Maize dwarf mosaic virus, strain-B. Phytopathology.

[bib0125] Goncalves M.C., Galdeano D.M., Maia I.D., Chagas C.M. (2011). Genetic variability of Sugarcane mosaic virus causing maize mosaic in Brazil. Pesqui. Agropecu. Bras..

[bib0130] Gonza´lez-Jara P., Atencio F.A., Martı´nez-Garcı´a B., Barajas D., Tenllado F., Dı´az-Ruı´z J.R. (2005). A single amino acid mutation in the plum pox virus helper component-proteinase gene abolishes both synergistic and RNA silencing suppression activities. Phytopathology.

[bib0135] Hadi B.A.R., Langham M.A.C., Osborne L., Tilmon K.J. (2011). Wheat streak mosaic virus on wheat: biology and management. Integ. J. Pest Manag..

[bib0140] Hansford C.G. (1935). Sugarcane diseases in Uganda. E.A. Agric. J..

[bib0145] Jensen S.G., Wysong D.S., Ball E.M., Higlem P.M. (1991). Seed transmission of maize chlorotic mottle virus. Plant Dis..

[bib0150] Johnson M., Zaretskaya I., Raytselis Y., Merezhuk Y., McGinnis S., Madden T.L. (2008). NCBI BLAST: a better web interface. Nucleic Acids Res..

[bib0155] Jones M.W., Redinbaugh M.G., Louie R. (2007). The Mdm1 locus and maize resistance to maize dwarf mosaic virus. Plant Dis..

[bib0160] Kagoda F., Gidoi R., Brian E., Isabirye B.E. (2016). Status of maize lethal necrosis in eastern Uganda. Afr. J. Agric. Res..

[bib0165] KEPHIS (2016). Kenya Plant Health Inspectorate Service (KEPHIS) Annual Report.

[bib0170] Kimura M. (1980). A simple method for estimating evolutionary rate of base substitutions through comparative studies of nucleotide sequences. J. Mol. Evol..

[bib0175] King A.M.Q., McCahon D., Slade W.R., Newman J.W.I. (1982). Recombination in RNA. Cell.

[bib0180] Kulkarni H.Y. (1973). Notes on east african plant virus diseases. East Afr. Agric. For. J..

[bib0185] Lemey P., Lott M., Martin D.P., Moulton V. (2009). Identifying recombinants in human and primate immunodeficiency virus sequence alignments using quartet scanning. BMC Bioinformatics.

[bib0190] Li Y.Q., Liu R.Y., Zhou T., Fan Z.F. (2013). Genetic diversity and population structure of Sugarcane mosaic virus. Virus Res..

[bib0195] Lim S., Yoon Y., Jang Y.W., Bae S., Lee Y.H., Lee B.C. (2018). First report of maize yellow mosaic virus on Zea mays in South Korea. Plant Dis..

[bib0200] Lommel S.A., Kendall T.L., Xiong Z., Nutter R.C. (1991). Identification of the Maize chlorotic mottle virus capsid protein cistron and characterization of its subgenomic messenger RNA. Virology.

[bib0205] Louie R. (1980). Sugarcane mosaic virus in Kenya. Plant Dis..

[bib0210] Lukanda M., Owati A., Ogunsanya P., Valimunzigha K., Katsongo K., Ndemere H., Kumar L.P. (2014). First report of Maize chlorotic mottle virus infecting maize in the Democratic Republic of the Congo. Plant Dis..

[bib0215] Mahuku G., Lockhart B., Wanjala B., Jones M., Kimunye J., Stewart L. (2015). Maize lethal necrosis (MLN), an emerging threat to maize-based food security in sub-Saharan Africa. Phytopathology.

[bib0220] Mahuku G., Wangai A., Sadessa K., Teklewold A., Wegary D., Adams (2015). First report of maize chlorotic mottle virus and maize lethal necrosis on maize in Ethiopia. Plant Dis..

[bib0225] Marenya P.P., Erenstein O., Prasanna B., Makumbi D., Jumbo M., Beyene Y. (2018). Maize lethal necrosis disease: evaluating agronomic and genetic control strategies for Ethiopia and Kenya. Agric. Syst..

[bib0230] Martin D.P., Williamson C., Posada D. (2005). RDP2: recombination detection and analysis from sequence alignments. Bioinformatics..

[bib0235] Martin D.P., Murrell B., Golden M., Khoosal A., Muhire B. (2015). RDP4, Detection and analysis of recombination patterns in virus genomes. Virus Evol..

[bib0240] Massawe D.P., Stewart L.R., Kamatenesi J., Asiimwe T., Redinbaugh M.G. (2018). Complete sequence and diversity of a maize-associated polerovirus in East Africa. Virus Genes.

[bib0245] Maynard Smith J. (1992). Analysing the mosaic structure of genes. J. Mol. Evol..

[bib0250] McDonald J. (1936). A revised list of plant diseases in Kenya colony. E. Afr. Agric. J..

[bib0255] Ministry of Agriculture (2017). Ministry of Agriculture Liv and Development Biannual Reports.

[bib0260] Nault L.R., Styer W.E., Coffey M.E., Gordon D.T., Negi L.S., Niblett C.L. (1978). Transmission of maize chlorotic mottle virus by chrysomelid beetles. Phytopathology..

[bib0265] Nutter R.C., Scheets K., Panganiban L., Lommel S.A. (1989). The complete nucleotide sequence of maize chlorotic mottle virus. Nucleic Acids Res..

[bib0270] Nyasani J.O., Subramanian S. (2012). Insect Vectors Associated With Transmission of MLV Viruses.

[bib0275] Ondov B., Bergman N., Phillippy A. (2011). Krona: interactive metagenomic visualization in a web browser. BMC Bioinformatics.

[bib0280] Ouma J., Bett E., Mbataru P. (2014). Drivers of adoption of improved maize varieties in moist transitional zone of Eastern Kenya. Journal of Economics and Sustainable Development.

[bib0285] Padidam M., Beachy R., Fauquet C.M. (1995). Classification and identification of geminiviruses using sequence comparisons. J. Virol..

[bib0290] Perez-Losada M., Arenas M., Galan J.C., Palero F., Gonzalez-Candelas F. (2015). Recombination in viruses: mechanisms, methods of study, and evolutionary consequences. Infect. Genet. Evol..

[bib0295] Posada D., Crandall K.A. (2001). Evaluation of methods for detecting recombination from DNA sequences: computer simulations. Proc. Natl. Acad. Sci. U.S.A..

[bib0300] Prasanna B.M. (2015). Maize lethal necrosis (MLN) in eastern Africa: tackling a major challenge. In: The African Seed.

[bib0305] Pruss G., Ge X., Shi X.M., Carrington J.C., Bowman, Vance V. (1997). Plant viral synergism: the potyviral genome encodes a broad-range pathogenicity enhancer that transactivates replication of heterologous viruses. Plant Cell.

[bib0310] Read D.A., Featherston J., Rees D.J.G. (2019). Molecular characterization of Morogoro maize-associated virus, a nucleorhabdovirus detected in maize (Zea mays) in Tanzania. Arch. Virol..

[bib0315] Riley E.A. (1960). A revised list of plant diseases in Tanganyika Territory. Mycol. Pap..

[bib0320] Salminen M.O., Carr J.K., Burke D.S., McCutchan F.E. (1995). Identification of breakpoints in intergenotypic recombinants of HIV type 1 by bootscanning. AIDS Res. Hum. Retrovirus..

[bib0325] Scheets K. (1998). Maize chlorotic mottle machlomovirus and wheat streak mosaic rymovirus concentrations increase in the synergistic disease corn lethal necrosis. Virology.

[bib0330] Semagn K., Beyene Y., Babu R., Nair S., Gowda M., Das B., Tarekegne A., Mugo S., Mahuku G., Worku M., Warburton M.L., Olsen M., Prasanna B.M. (2015). Quantitative trait loci mapping and molecular breeding for developing stress resilient maize for sub-Saharan Africa. Crop Sci..

[bib0335] Sergey N., Dmitry M., Anton K., Pavel A. (2017). Pevzner metaSPAdes: a new versatile metagenomic assembler. Genome Res..

[bib0340] Stenger D.C., Young B.A., Qu F., Morris T.J., French R. (2007). Wheat streak mosaic virus lacking helper component-proteinase is competent to Produce Disease Synergism in double Infections with maize chlorotic mottle virus. Phytopathology..

[bib0345] Stewart L.R., Teplier R., Todd J.C., Jones M.W., Cassone B.C., Wijeratne S., Wijeratne A., Redinbaugh M.G. (2014). Viruses in maize and Johnsongrass in southern Ohio. Phytopathol..

[bib0350] Stewart L.R., Willie K., Wijeratne S., Redinbaugh M.G., Massawe D. (2017). Johnsongrass mosaic virus contributes to maize lethal necrosis in East Africa. Plant Dis..

[bib0355] Syller J. (2012). Facilitative and antagonistic interactions between plant viruses in mixed infections. Mol. Plant Pathol..

[bib0360] Tamura K., Stecher G., Peterson D., Filipski A., Kumar S. (2013). MEGA6: molecular evolutionary genetics analysis version 6.0. Mol. Biol. Evol..

[bib0365] USDA/FAS Grain Reports (2015). Global Agricultural Information Network.

[bib0370] Valli A., López-Moya J.J., García J.A. (2007). Recombination and gene duplication in the evolutionary diversification of P1 proteins in the family Potyviridae. J. Gen. Virol..

[bib0375] Varanda C.M.R., Nolasco G., Clara M.I., Félix M.R. (2014). Genetic diversity of the coat protein of olive latent virus 1 isolates. Arch. Virol..

[bib0380] Wallace G. (1937). A list of plant diseases of economic importance in Tanganyika Territory. E. Afr. Agric. J..

[bib0385] Wamaitha J.M., Deepti N., Solomon M., Francesca S., Anne W., Joyce N., Timothy A.H., Bramwel W.W., Mark W., Tanui L., Appolinaire D., Hernan G. (2018). Metagenomic analysis of viruses associated with maize lethal necrosis in Kenya. Virol. J..

[bib0390] Wang Q., Zhang C., Wang C., Qian Y., Li Z., Jian Hong J., Zhou X. (2017). Further characterization of Maize chlorotic mottle virus and its synergistic interaction with Sugarcane mosaic virus in maize. Sci. Rep..

[bib0395] Wangai A.W., Redinbaugh M.G., Kinyua Z.M., Mahuku G., Sheets K., Jeffers D. (2012). First report of Maize chlorotic mottle virus and maize lethal necrosis in Kenya. Plant Dis..

[bib0400] Xie X., Chen W., Fu Q., Zhang P., An T., Cui A. (2016). Molecular variability and distribution of sugarcane mosaic virus in Shanxi. China. PLoS ONE.

